# ProNGF/p75NTR Axis Drives Fiber Type Specification by Inducing the Fast-Glycolytic Phenotype in Mouse Skeletal Muscle Cells

**DOI:** 10.3390/cells9102232

**Published:** 2020-10-02

**Authors:** Valentina Pallottini, Mayra Colardo, Claudia Tonini, Noemi Martella, Georgios Strimpakos, Barbara Colella, Paola Tirassa, Sabrina Di Bartolomeo, Marco Segatto

**Affiliations:** 1 Department of Science, University of Rome “Roma Tre”, 00146 Rome, Italy; valentina.pallottini@uniroma3.it (V.P.); claudia.tonini@uniroma3.it (C.T.); 2 Department of Biosciences and Territory, University of Molise, 86090 Pesche (IS), Italy; m.colardo@studenti.unimol.it (M.C.); n.martella@studenti.unimol.it (N.M.); b.colella@studenti.unimol.it (B.C.); sabrina.dibartolomeo@unimol.it (S.D.B.); 3 Institute of Biochemistry and Cell Biology, National Research Council (CNR), 00015 Rome, Italy; georgios.strimpakos@cnr.it (G.S.); paola.tirassa@cnr.it (P.T.)

**Keywords:** differentiation, Duchenne muscular dystrophy, metabolism, myotubes, nerve growth factor, neurotrophins, p75 NTR, satellite cells, skeletal muscle

## Abstract

Despite its undisputable role in the homeostatic regulation of the nervous system, the nerve growth factor (NGF) also governs the relevant cellular processes in other tissues and organs. In this study, we aimed at assessing the expression and the putative involvement of NGF signaling in skeletal muscle physiology. To reach this objective, we employed satellite cell-derived myoblasts as an in vitro culture model. In vivo experiments were performed on Tibialis anterior from wild-type mice and an mdx mouse model of Duchenne muscular dystrophy. Targets of interest were mainly assessed by means of morphological, Western blot and qRT-PCR analysis. The results show that proNGF is involved in myogenic differentiation. Importantly, the proNGF/p75NTR pathway orchestrates a slow-to-fast fiber type transition by counteracting the expression of slow myosin heavy chain and that of oxidative markers. Concurrently, proNGF/p75NTR activation facilitates the induction of fast myosin heavy chain and of fast/glycolytic markers. Furthermore, we also provided evidence that the oxidative metabolism is impaired in mdx mice, and that these alterations are paralleled by a prominent buildup of proNGF and p75NTR. These findings underline that the proNGF/p75NTR pathway may play a crucial role in fiber type determination and suggest its prospective modulation as an innovative therapeutic approach to counteract muscle disorders.

## 1. Introduction

Neurotrophins are a class of trophic factors particularly known for their indisputable role in regulating the survival, differentiation and plasticity of nerve cells [1]. This family of growth factors consists of nerve growth factor (NGF), brain-derived neurotrophic factor (BDNF), neurotrophin-3 (NT-3) and neurotrophin-4 (NT-4). Neurotrophin signaling is mediated by two different kinds of receptors: the tropomyosin-related kinase (Trk) receptors and the p75 neurotrophin receptor (p75NTR) [2]. Both BDNF and NT-4 bind with high affinity TrkB, whereas NGF and NT-3 activate TrkA and TrkC, respectively. In contrast, p75NTR unselectively binds all of the neurotrophins with low affinity [3]. Neurotrophins are initially synthetized as pro-neurotrophin precursors, which may undergo post-translational processing to generate mature proteins. Interestingly, it is now well established that pro-neurotrophins also display biological activity: for instance, proNGF and proBDNF bind and activate p75NTR. The functional roles promoted by p75NTR may vary in dependence of the cell context, the presence of specific adapter proteins, the concurrent expression of Trk receptors, and the balance between the mature neurotrophin and its relative precursor form [4].

In addition to the central nervous system, accumulating evidence suggests that neurotrophins may exert pleiotropic effects in a broader range of cell types and tissues. Skeletal muscle has attracted increasing interest, as this tissue provides a considerable amount of neurotrophins, especially throughout development [1]. Furthermore, muscle cells express different neurotrophin receptors, suggesting that skeletal muscle is responsive to neurotrophins [5]. For instance, the lack of NT-3 or its receptor TrkC dramatically reduces muscle spindles, reflecting severe movement alterations [6,7]. The disruption of NT-4 signaling leads to the disassembly of postsynaptic acetylcholine receptor (AChR) clusters, thus affecting neurotransmission fatigue resistance [8]. The BDNF pathway has been particularly characterized in skeletal muscle, and a crucial involvement of this neurotrophin has been highlighted in neuromuscular junction structure, muscle regeneration, and myogenesis [9,10,11]. Interestingly, a very recent report indicated that BDNF could act as a autocrine/paracrine factor to transcriptionally mediate the fast/glycolytic program in muscle fibers [12]. Among neurotrophins, NGF seems to be particularly involved in muscle regeneration, although experimental evidence is still fragmented. It has been observed that anti-NGF transgenic mice show muscle atrophy, characterized by the appearance of centrally nucleated fibers and inflammatory infiltrate reminiscent of a dystrophic phenotype [13,14]. NGF immunoreactivity is particularly confined to regenerating fibers, suggesting that this neurotrophin may trigger and support muscle regeneration [13]. The majority of experimental findings underlie that NGF exerts biological effects on skeletal muscle by binding to p75NTR, whereas no detectable levels or a very low expression have been described for TrkA [15,16]. Notably, NGF induces myogenic differentiation and muscle repair through p75NTR, possibly by modulating RhoA and NFkB signaling [16,17,18]. Conversely, other evidence supports the notion that persistent stimulation with NGF significantly represses myogenic differentiation in p75NTR-overexpressing myoblasts [19]. Despite these data, information about the role of the NGF/p75NTR pathway is still limited and not conclusive. For instance, the employment of immunohistochemical and transcriptomic techniques in previous studies does not allow to identify the putative contribution of mature NGF or proNGF in skeletal muscle physiology. Additionally, no reports to date have comprehensively investigated the prospective role of the NGF/p75NTR pathway in the physiological regulation of skeletal muscle metabolism. Here, we provide the first evidence that proNGF influences fiber type specification by promoting a faster and more glycolytic phenotype in myotubes from satellite cell-derived myoblasts (SCDMs). This event is mediated by p75NTR, as its inhibition by the small-molecule ligand LM11A-31 completely blocks the outcomes induced by proNGF administration. Furthermore, proNGF and p75NTR expression are increased in dystrophic muscles, suggesting that this pathway may contribute to metabolic changes observed in Duchenne muscular dystrophy (DMD).

## 2. Materials and Methods

### 2.1. Satellite Cell Isolation, Culturing and Treatments

Satellite cell isolation was performed by following the previously described protocol [20]. After isolation, the satellite cells were grown on gelatin-coated dishes in a proliferation medium (PM) composed of DMEM, 20% FBS, 3% chicken embryo extract (prepared in Hank’s Balanced Solution), and 2.5 ng/mL bFGF (Peprotech, Rocky Hill, NJ, USA), as already reported [21]. To induce myotube differentiation, satellite cells were seeded at high density and PM was replaced with a differentiation medium (DM), consisting of DMEM containing 2% horse serum (HS). Complete differentiation was achieved after 72 h. The beginning and duration of the treatment with 500 ng/mL of NGF/proNGF neutralizing antibody (Alomone labs, Jerusalem, Israel; ALM-006), 100 ng/mL of recombinant mouse proNGF (Alomone labs, Jerusalem, Israel; N-250), and/or 100 nM of LM11A-31 (Sigma Aldrich, Milan, Italy; SML0664) is indicated in the figure legends.

### 2.2. Animals

All the experiments involving the use of laboratory animals were approved by the Italian Ministry of Health and carried out in compliance with the guidelines of the Italian Ministry of Health (according to Legislative Decree 116/92), the Directive 2010/63/EU of the European Parliament and the Council on the protection of animals used for scientific purposes (Protocols 3/2014 and 791/2018). Animals were allocated in the animal facility in a temperature-controlled room [22]. Notably, WILD-TYPE (WT) mice (C57BL/10ScSnJ) and *mdx* mice (C57BL/10ScSn-Dmdmdx/J) were housed in groups of five under controlled temperature (20 ± 1 °C), humidity (55 ± 10%), and illumination (lights on for 12 h per day, from 7:00 a.m. to 07:00 p.m.). Food and water were provided ad libitum. Tubes for tunneling and nesting materials (paper towels) were placed in every cage as environmental enrichment. WT and *mdx* were sacrificed at 3 months of age; Tibialis anterior (TA) muscles were immediately dissected out and subsequently processed for biochemical and morphological analysis.

### 2.3. Total Lysate Preparation and Western Blot Analysis

Cultured cells and TA muscles from WT and *mdx* mice were lysed in sample buffer (0.125 M Tris-HCl containing 10% SDS, protease inhibitor cocktail, pH 6.8) by sonication (duty cycle 20%, output 3) as previously described [23,24]. Subsequently, after the addition of the Laemmli buffer and denaturation at 95 °C for 5 min, the samples (twenty micrograms of proteins) were resolved on SDS-PAGE [25]. Protein transfer onto a nitrocellulose membrane was carried out by using the standard protocol for trans-blot turbo transfer system (Biorad Laboratories, Hercules, CA, USA), as previously indicated [26]. Nitrocellulose membrane was then blocked at room temperature for 1 h with 5% no-fat dry milk in Tris-buffered saline (138 mM NaCl, 27 mM KCl, 25 mM Tris-HCl, 0.05% Tween-20, pH 6.8), and incubated at 4 °C overnight with the following primary antibodies: anti-p75NTR (Santa Cruz Biotechnology, Dallas, TX, USA; sc-271708, dilution 1:1000), anti-NGF (Santa Cruz Biotechnology, Dallas, TX, USA; sc-365944, dilution 1:1000), anti-slowMyHC clone NOQ7.5.4D (Sigma-Aldrich, Milan, Italy; SAB4200670, dilution 1:5000), anti-fastMyHC clone MY-32 (Sigma-Aldrich, Milan, Italy; M4276, dilution 1:10,000), anti-SREBP-1 (Santa Cruz Biotechnology, Dallas, TX, USA; sc-8984, dilution 1:1000), anti-Cytochrome C (Santa Cruz Biotechnology, Dallas, TX, USA; sc-13156, dilution 1:500), anti-COXIV (Santa Cruz Biotechnology, Dallas, TX, USA; sc-69359, dilution 1:500), anti-alpha-Tubulin (Sigma-Aldrich, Milan, Italy; T6199, dilution 1:10,000), anti-phosphoCREB (Santa Cruz Biotechnology, Dallas, TX, USA; sc-7978, dilution 1:1000), anti-phosphoAMPK (Santa Cruz Biotechnology, Dallas, TX, USA; sc-33524, dilution 1:1000), anti-PGC-1α (Santa Cruz Biotechnology, Dallas, TX, USA; sc-13067, dilution 1:500), anti-Necdin (Abcam, ab18554, Cambridge, UK; dilution 1:1000), anti-H3 (Santa Cruz Biotechnology, Dallas, TX, USA; sc-10809, dilution 1:1000), and anti-GAPDH (Santa Cruz Biotechnology, Dallas, TX, USA; sc-32233, dilution 1:11000). The day after, the membranes were probed for 1 h with horseradish peroxidase-conjugated secondary IgG antibodies (Bio-Rad Laboratories, Milan, Italy). Protein-bound antibodies were visualized through clarity ECL Western blotting (Bio-Rad Laboratories, Milan, Italy; #1705061), and chemiluminescence was acquired by using ChemiDoc MP system (Bio-Rad Laboratories). The densitometric evaluation of images derived from Western blotting was then performed by using ImageJ (National Institutes of Health, Bethesda, MD, USA) software for Windows. Alpha-Tubulin was chosen as the internal control for protein loading. Recorded values were obtained from the ratio between the arbitrary units of the protein band and the respective housekeeping protein. The specificity of anti-NGF (E-12, sc- sc-365944) to bind both proNGF and mature NGF was tested by performing Western blot analysis on the hippocampi from wild-type and mdx mice, chosen as the positive control for NGF species detection (Figure S1).

### 2.4. Cell Fractionation

Cytosolic and nuclear fractionation from cultured cells and TA muscles were performed as previously reported [27].

### 2.5. Morphological Analysis

Immunofluorescence analysis on satellite cells or differentiated myotubes was carried out by slightly modifying the previously described protocol [28]. Briefly, the cells were cultured on gelatin-coated glass coverslips and after treatment, fixed in 4% paraformaldehyde (PFA) in PBS for 10 min, and permeabilized with 0.3% Triton X-100 in PBS for 30 min. Cells were then blocked at room temperature with 10% normal goat serum (NGS) in PBS for 1 h, and probed overnight with anti-p75NTR antibody (Santa Cruz Biotechnology, Dallas, TX, USA; sc-271708, dilution 1:100) or anti-MyHC (MF20). Cells were subsequently washed three times with PBS and incubated for 1 h in the dark with goat anti-rabbit secondary antibody Alexa Fluor 555 (ThermoFisher Scientific, Milan, Italy; A27039) or goat anti-mouse secondary antibody Alexa Fluor 488 (ThermoFisher Scientific, Milan, Italy; A28175). Finally, coverslips were mounted with Vectashield Antifade mounting medium with DAPI (Vector Laboratories, San Francisco, CA, USA; H-1200) for nuclear counterstaining. Immunofluorescent cells were observed using an Axio Imager Z2 microscope (Carl Zeiss, Milan, Italy) equipped with a charge-coupled device (CCD) camera controlled by the ISIS software (MetaSystems, Altlussheim, Germany). For myotube differentiation analysis, the fusion index was calculated as the percentage of nuclei into fused myotubes out of the total nuclei per field.

To perform immunohistochemistry and immunofluorescence on the WT and *mdx* mice, OCT frozen TA muscles were used. Notably, 10 μm-thick transverse sections were cut on cryostat and collected on Superfrost Plus slides (BioOptica, Milan, Italy). Slices were post-fixed by immersion in 4% PFA for 10 min, and permeabilized with 0.3% Triton X-100 in PBS for 1 h. Slices were also blocked for 1 h with 10% NGS in PBS, and then probed overnight with primary antibody against p75NTR (Santa Cruz Biotechnology, Santa Cruz, CA, USA; sc-271708, dilution 1:100). The next day, sections dedicated to immunofluorescence were processed as described above. Otherwise, TA slices were sequentially incubated with biotinylated goat anti-mouse IgG (Vector Laboratories, San Francisco, CA, USA) and with the Vectastain Elite ABC-HRP kit (Vector Laboratories, San Francisco, CA, USA; PK-6100) for immunohistochemistry. Immunocomplexes were visualized by using ImmPACT DAB peroxidase (HRP) substrate (Vector Laboratories, San Francisco, CA, USA; SK-4105). Sections were dehydrated in ascending alcohol series and mounted with Eukitt (Kindler GmbH & Co, Bobingen, Germany). Pictures of muscle slices were acquired under a Leica CTR6000 microscope (Leica Microsystems, Wetzlar, Germany) equipped with Leica DFC480 camera for bright-field visualization. Images were captured using the Leica Application Suite System and the files were converted in TIFF format.

### 2.6. EdU Staining

SCDMs were isolated and kept in the proliferation medium. After 45 h, EdU (5-ethynyl-2′-deoxyuridine) was added into the culture medium at the concentration of 10 µM, and the SCDMs were cultured for an additional three hours. EdU staining was then carried out by using Click-iT EdU Imaging Kit (Thermo Fisher Scientifc, Milan, Italy) according to the manufacturer’s instruction. DAPI was used to counterstain the nuclei. The percentage of EdU+ cells was calculated as the number of EdU+ nuclei divided by the total number of nuclei per field.

### 2.7. RNA Extraction and Real-Time PCR

Total RNA from cells or frozen TA was extracted using TRIzol Reagent (Sigma-Aldrich, Milan, Italy) according to the manufacturer’s instructions. After DNAse treatment (Ambion, Thermo Fisher Scientific, Milan, Italy), RNA was purified with an RNA clean up Kit (Zymo, Italy). Subsequently, the RNA was reverse transcribed to cDNA through High-Capacity cDNA Reverse Transcription Kit (Applied Bio-System, Foster City, CA, USA), and employed for qPCR analysis. Primers used in qRT-PCR are reported in Table S1. All the oligonucleotides used were optimized for real-time amplification in a standard curve amplification (>98% for each pair of oligos) and the generation of the correct amplicon was verified by melting curve analysis. Each biological sample was tested in triplicate using SYBR green IQ reagent (Bio-Rad Laboratories, Milan, Italy), with CFX Connect detection system (Bio-Rad Laboratories, Milan, Italy). *Hprt* was selected as the reference gene.

### 2.8. Co-Immunoprecipitation

Co-immunoprecipitation experiments on nuclear extracts from WT and *mdx* TA muscles were performed by following the previously described protocol with modifications [29]. Briefly, 1 mg of proteins were incubated with 10 μg of p75NTR antibody (Sigma-Aldrich, Milan, Italy; 07-476). Nuclear fractions were probed with the antibody for 1 h at 4 °C, subsequently 50 μL of protein A-agarose was added for 1 h at 4 °C. Samples were then centrifuged (50,000× *g* for 15 min), and the pellet was gently washed three times with washing buffer to eliminate non-specific binding. Immunoprecipitated proteins were denatured in Laemmli buffer by heat treatment and separated in SDS-PAGE. Proteins were electrophoretically transferred onto nitrocellulose and then probed overnight at 4 °C with anti-p75NTR (Santa Cruz Biotechnology, sc-271708, dilution 1:1000) or anti-Necdin (Abcam, ab18554, dilution 1:1000) antibodies. For necdin visualization, secondary anti-rabbit IgG HRP “True blot” (Rockland Immunochemicals) was employed.

### 2.9. Statistical Analysis

All the experimental data are expressed as the means ± SD (standard deviation). When two experimental groups were compared, an unpaired Student’s *t*-test was used. Conversely, one-way analysis of variance (ANOVA) followed by the Tukey’s post hoc test was used to compare three or more experimental groups. Two-way ANOVA was employed when two independent variables (time and treatment) were present. The values of *p* < 0.05 were considered to indicate a statistically significant difference. Statistical analysis was performed using GRAPHPAD INSTAT3 (GraphPad, La Jolla, CA, USA) for Windows.

## 3. Results

### 3.1. Satellite Cell-Derived Myoblasts Express proNGF and p75NTR

**Figure 1:**
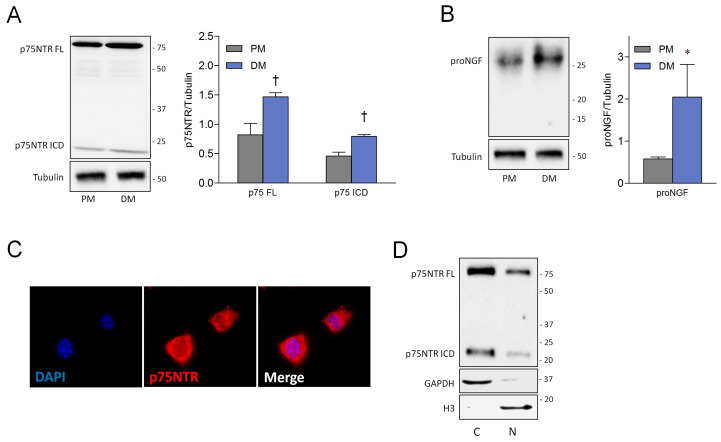
ProNGF and p75NTR are expressed in satellite cell-derived myoblasts (SCDMs) under both proliferating and differentiating conditions. (**A**) Western blot and densitometric analysis of p75NTR full length (p75NTR FL) and p75NTR intracellular domain (p75NTR ICD) in the total lysates of SCDMs cultured in the proliferation medium (PM) and differentiation medium (DM) for 24 h, derived from three independent experiments. (**B**) Western blot and densitometric analysis of proNGF in the total lysates of SCDMs cultured as in A. N = 3 independent experiments. Tubulin was employed as a loading control. (**C**) p75NTR immunofluorescence staining (red) in SCDMs kept in DM for 24 h. DAPI was used to counterstain nuclei. N = 3 independent experiments. (**D**) Western blot of p75NTR performed on nuclear extracts from SCDMs, 24 h after DM addition. H3 and GAPDH were used as protein loading controls and as nuclear and cytoplasmic markers, respectively. N = 3 independent experiments. Values are represented as the mean ± SD. Statistical analysis was performed by using the Student’s *t* test. * *p* < 0.05; ^†^ *p* < 0.01. C = cytosolic fraction; N = nuclear fraction.

To start, we investigated the expression of NGF species and p75NTR in SCDMs. According to previous reports [16], we found that full-length p75NTR (p75NTR FL) is expressed in SCDMs cultured in proliferation medium (PM), and its expression further increases upon 24 h of differentiation medium (DM) (Figure 1A). It has been observed that p75NTR may undergo proteolytic cleavage to yield a cytoplasmic/nuclear fragment capable of signaling properties [30]. Interestingly, we noted for the first time that p75NTR intracellular domain (p75NTR ICD) was produced in both proliferating and differentiating SCDMs. Concerning NGF species, proNGF (26 kDa) was found to be expressed at appreciable levels in proliferating SCDMs, and its levels significantly increased to 24 h after DM addition. Notably, mature NGF (13.2 kDa) was not detected in both proliferating and differentiating cells (Figure 1B). The p75NTR expression was also confirmed by means of immunofluorescence analysis. Similarly to what was already observed in neurons [31], p75NTR was primarily localized to the cytoplasm and plasma membrane. However, immunopositivity was also noted at the nuclear level (Figure 1C). Nuclear/cytosol fractionation experiments corroborated this observation, demonstrating that both p75NTR FL and p75NTR ICD are expressed at the nuclear level, although at lower amounts if compared to the cytosolic compartment (Figure 1D). Thus, proNGF is the predominant form of NGF, and p75NTR is diffusely expressed, suggesting that SCDMs may be responsive to the proNGF/p75NTR pathway.

### 3.2. Evaluation of proNGF Effects on SCDM Proliferation and Differentiation

**Figure 2:**
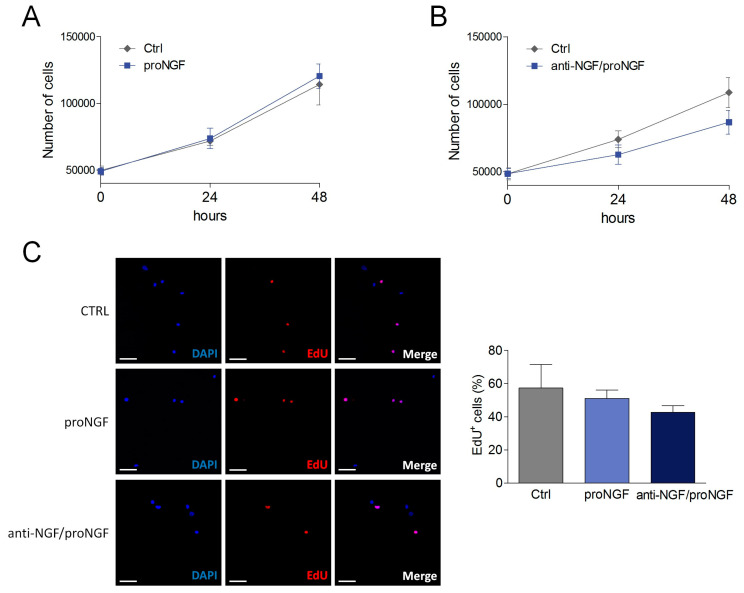
Effect of proNGF administration and anti-NGF/proNGF addition on SCDM proliferation. (**A**) SCDMs were cultured in a proliferation medium and treated with proNGF (100 ng/mL) up to 48 h. Cells were counted at both 24 and 48 h to evaluate cell growth. (**B**) SCDM cell growth was evaluated as in A, in both control cells and SCDMs treated with neutralizing anti-NGF/proNGF antibody (500 ng/mL) for 48 h. (**C**) EdU staining and the ratio of EdU^+^ cells from proliferating SCDMs, kept in a proliferation medium in the presence or not of proNGF (100 ng/mL) or anti-NGF/proNGF (500 ng/mL) for 48 h. DAPI was used to counterstain the nuclei. Three images for each experimental group were captured under a fluorescence microscope, and then analyzed to evaluate the percentage of EdU^+^ cells. All the experiments consist of at least three biological replicates. Scale bar: 50 μm. Values are expressed as the mean ± SD. For cell proliferation, statistical analysis was performed by using two-way ANOVA, whereas the ratio of EdU^+^ cells was analyzed by one-way ANOVA followed by Tukey’s post hoc test.

To investigate the biological activity of proNGF on SCDMs, proliferating and differentiating cells were cultured in the presence of a neutralizing NGF/proNGF antibody, or of exogenous proNGF. Neither proNGF nor anti-NGF/proNGF administration significantly affected SCDM proliferation (Figure 2A,B). These results were corroborated by the analysis of EdU incorporation, showing that the percentage of EdU^+^ cells was not dissimilar among the analyzed experimental groups (Figure 2C).

**Figure 3:**
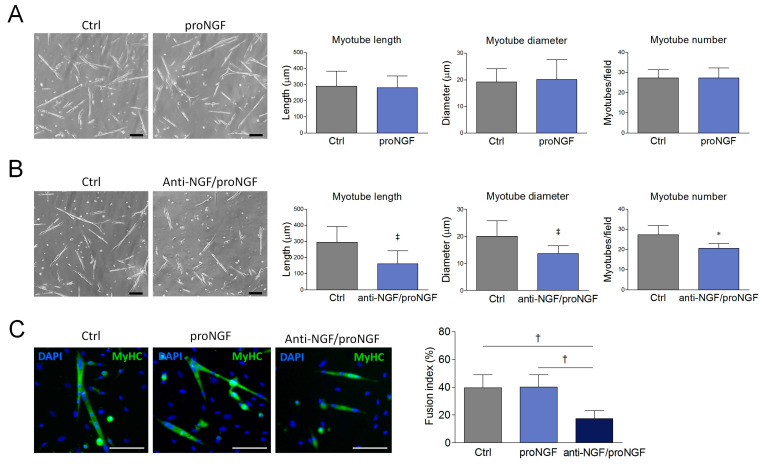
Effect of proNGF administration and anti-NGF/proNGF addition on SCDM differentiation. (**A**) SCDMs were allowed to differentiate into myotubes in the differentiation medium (DM), in presence or not of proNGF (100 ng/mL) for 72 h. Cells were observed with bright field microscopy and three images for every single experiment were captured. (**B**) SCDMs were treated with anti-NGF/proNGF (500 ng/mL) and induced to differentiate in DM for 72 h. Three images for each experimental group were captured under a brightfield microscope, and then analyzed to evaluate myotube length, myotube diameter and myotube number. (**C**) SCDMs were induced to differentiate in the presence of proNGF (100 ng/mL) or anti-NGF/proNGF (500 ng/mL) for 72 h. Once differentiation was completed, the cells were fixed in paraformaldehyde (PFA) 4% and incubated overnight with anti-MyHC (green) to evaluate the fusion index by immunofluorescence. DAPI (blue) was used to counterstain the nuclei. Fusion index was calculated as the percentage of nuclei incorporated into myotubes on the total nuclei present in each field. All the experiments consisted of at least three biological replicates. Scale bars: 100 μm. Values are expressed as the mean ± SD. Statistical analysis was performed by using the Student’s *t* test. * *p* < 0.05; ^†^ *p* < 0.01; ^‡^ *p* < 0.001.

ProNGF treatment was also unable to induce significant changes in SCDM differentiation into myotubes (Figure 3A,C). Conversely, the addition of anti-NGF/proNGF impaired myogenic differentiation, as observable by the significant decrease in myotube number and by the reduction in myotube length and diameter (Figure 3B). Importantly, the fusion index, a widely employed parameter to quantify myogenic differentiation in cell culture [32], was deeply affected upon neutralizing antibody incubation (Figure 3C). Taken together, these results suggest that the addition of exogenous proNGF does not affect SCDM differentiation, since endogenous proNGF is already sufficient to induce a maximal effect.

### 3.3. ProNGF Promotes a Fast Phenotype during SCDM Differentiation

**Figure 4:**
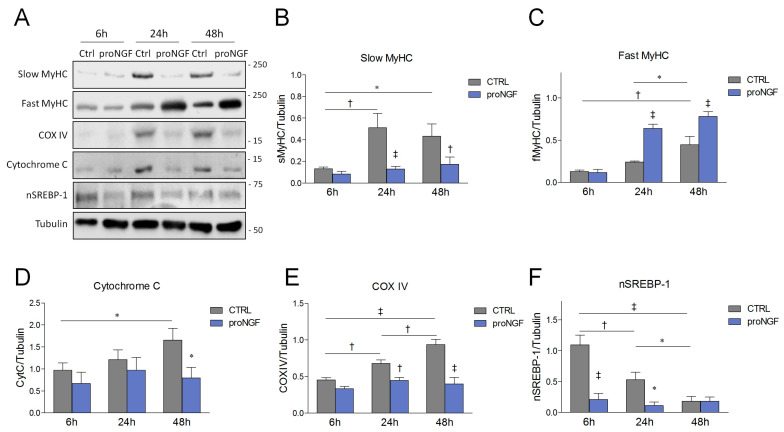
Effects of proNGF administration on the slow/oxidative markers during SCDM differentiation. (**A**) Western blot and densitometric analysis of (**B**) slow myosin heavy chain (slow MyHC), (**C**) fast MyHC), (**D**) cytrochrome C, (**E**) COX IV and (**F**) a nuclear and transcriptionally active fragment of SREBP-1 (nSREBP-1). SCDMs were allowed to differentiate in the differentiation medium, and treated with vehicle (PBS) or proNGF (100 ng/mL). Then, 6, 24 and 48 h after the induction of the differentiation, the cells were harvested and the total lysates were performed to evaluate the protein expression by Western blot. Tubulin was chosen as the housekeeping protein to normalize the protein loading. Data are expressed as the mean ± SD, and derived from three independent experiments. Statistical analysis was performed by using two-way ANOVA. * *p* < 0.05; ^†^ *p* < 0.01; ^‡^ *p* < 0.001.

Skeletal muscle is composed by heterogeneous fibers characterized by the different expression of myosin heavy chain (MyHC) isoforms. Fibers expressing the slower MyHC isoforms (MyHC I and MyHC IIa) have greater resistance to fatigue, are rich in mitochondria and mainly rely on oxidative phosphorylation to produce energy. Differently, fibers expressing faster MyHC (MyHC IIx, MyHC IIb) show reduced mitochondrial content and mostly take advantage of glycolytic metabolism [33,34]. In order to analyze whether proNGF treatment could modulate fiber type specification during myogenic differentiation, SCDMs were allowed to differentiate in the DM in the presence or absence of proNGF, and prospective protein changes were analyzed at 6, 24 and 48 h of differentiation. The addition of exogenous proNGF dramatically counteracted the rise in slow myosin heavy chain MyHC at 24 and 48 h (Figure 4A,B). On the other hand, this pro-neurotrophin was able to significantly enhance the expression of fast MyHC 24 and 48 h after the induction of differentiation (Figure 4A,C). Interestingly, proNGF treatment also prevented the time-dependent upregulation of the oxidative markers cytochrome C and COX IV (Figure 4A,D,E). We then evaluated the expression of the sterol regulatory element-binding protein 1 (SREBP-1). SREBP-1 proteins are master transcription factors mainly devoted to fatty acid biosynthesis. Notably, it has been observed that SREBP-1 supports oxidative metabolism by activating fatty acid production [35]. In our experimental conditions, the expression of the nuclear and transcriptionally active fragment of SREBP-1 (nSREBP-1) was sustained 6 h after DM addition, and gradually decreased along the time course. In contrast, proNGF administration maintained nSREBP-1 at low and constant levels throughout the experimental time points (Figure 4A,F), supporting the notion that proNGF drives SCDM differentiation into a faster and less oxidative phenotype.

**Figure 5:**
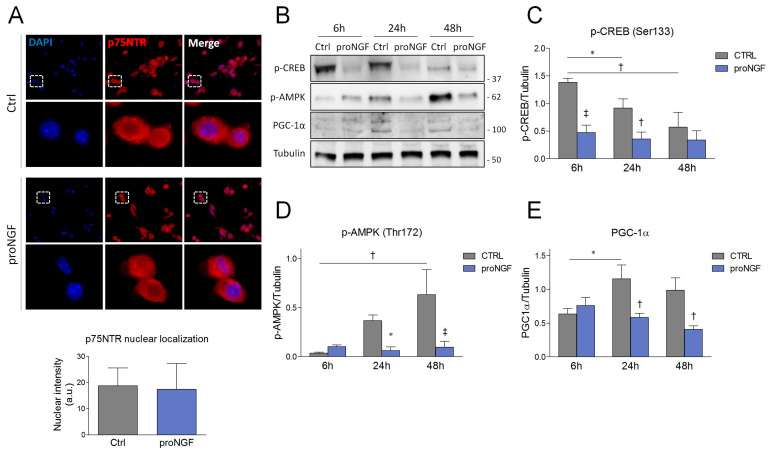
The evaluation of p75NTR localization and signaling cascades involved in the regulation of muscle fiber metabolism in differentiating SCDMs treated with proNGF. (**A**) SCDMs were induced to differentiate into the differentiation medium, and were treated with vehicle (PBS) or proNGF (100 ng/mL). Six hours after treatment, SCDMs were fixed and processed to carry out immunofluorescence analysis for p75NTR (red). DAPI was employed to visualize the nuclei. Nuclear intensity derived from p75NTR immunopositivity was analyzed by ImageJ and reported as arbitrary units (a.u.). N = 3. (**B**) Western blot and the densitometric analysis of (**C**) CREB phosphorylation at Ser133 (p-CREB), (**D**) AMPK phosphorylation at Thr172 (p-AMPK) and (**E**) PGC-1α. Differentiating SCDMs were treated with vehicle (PBS) or proNGF (100 ng/mL). Cells were collected at 6, 24, and 48 h and protein expression by means of Western blot was assessed with the total lysates. Tubulin was used as the housekeeping protein to normalize the protein loading. Results are expressed as the mean ± SD, and were obtained from three independent experiments. Statistical analysis was performed by using two-way ANOVA. * *p* < 0.05; ^†^ *p* < 0.01; ^‡^ *p* < 0.001.

Subsequently, we checked for putative molecular mechanisms explaining the proNGF-induced metabolic shift. Experimental evidence highlights that both p75NTR FL and p75ICD may translocate to the nucleus and regulate gene transcription [30,36]. Furthermore, NGF/proNGF stimulation enhances p75NTR processing and translocation into the nuclear compartment [37]. Thus, we hypothesized that proNGF-mediated alterations could be ascribable to changes in p75NTR nuclear translocation. However, no differences were observed between the differentiating SCDMs treated with vehicle or proNGF (Figure 5A), excluding the direct involvement of p75NTR in nuclear dynamics.

Fiber type determination and oxidative capacity are controlled by critical signaling proteins which converge on gene transcription to coordinately regulate the expression of slow-twitch muscle proteins and of metabolic enzymes. Among these regulators, AMP-activated protein kinase (AMPK) and cAMP response element-binding protein (CREB) are considered the most important players in muscle plasticity, as their activation is able to efficiently evoke a slow/oxidative myogenic program [38,39]. In light of this observation, we assessed whether proNGF administration could alter CREB and AMPK activity during myogenesis. Western blot analysis revealed that CREB activating phosphorylation at ser133 (p-CREB) was markedly sustained at 6 h, and progressively decreased during differentiation. On the contrary, when proNGF was added to DM, p-CREB was nearly suppressed in all the experimental time points taken into consideration (Figure 5B,C).

AMPK activating phosphorylation at thr172 was also modulated during SCDM differentiation: in particular, p-AMPK levels significantly increased during differentiation. In contrast, proNGF treatment completely prevented the activation of this kinase (Figure 5B,D). Literature data suggest that AMPK influences muscle metabolism by synergizing with CREB activity to regulate the expression and function of peroxisome proliferator-activated receptor-γ coactivator-1α (PGC-1α) [38,39]. Thus, PGC-1α protein levels were checked. Coherently with the suppression in p-CREB and p-AMPK levels, PGC-1α elevation was reduced upon proNGF treatment in differentiating SCDMs (Figure 5B,E). When evaluated as a whole, the collected data indicated that proNGF administration promoted the induction of fast MyHC during myogenic differentiation and concurrently, downregulated the expression of slow MyHC and of oxidative markers. In addition, these events are accompanied by the suppression of proteins controlling the slow/oxidative program, such as CREB, AMPK and PGC-1α.

### 3.4. ProNGF Induces Slow-to-Fast Transition in Myotubes

**Figure 6:**
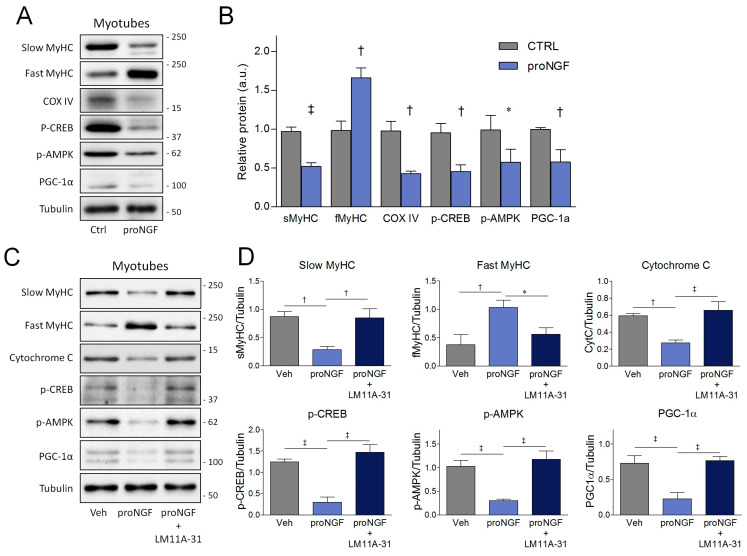
Effects of proNGF/p75NTR axis on slow/oxidative markers in differentiated myotubes. (**A**) Western blots and (**B**) densitometric analysis of the slow myosin heavy chain (sMyHC), fast MyHC (fMyHC), COX IV, CREB phosphorylation (p-CREB), AMPK phosphorylation (p-AMPK) and PGC-1α in the myotubes treated with proNGF. (**C**) Western blots and (**D**) densitometric analysis of slow myosin heavy chain (sMyHC), fast MyHC (fMyHC), cytochrome C, CREB phosphorylation (p-CREB), AMPK phosphorylation (p-AMPK) and the PGC-1α of myotubes derived from terminally differentiated SCDMs treated with proNGF or proNGF + LM11A-31. SCDMs were allowed to reach terminal differentiation into myotubes for 72 h. Subsequently, mature myotubes were treated with vehicle (PBS), proNGF (100 ng/mL) and proNGF (100 ng/mL) + LM11A-31 (100 nM) for 48 h. Tubulin was employed as the loading control. Results are expressed as the mean ± SD and were obtained from three independent experiments. Statistical analysis was performed by using the Student’s *t* test in panel B, whereas one-way ANOVA was used to calculate statistical significance in panel D. * *p* < 0.05; ^†^ *p* < 0.01; ^‡^ *p* < 0.001.

Our results suggest that proNGF drives the induction of a fast-twitch phenotype during SCDM differentiation. Therefore, our next goal was to evaluate whether proNGF administration was also able to induce a slow-to-fast fiber shift in differentiated myotubes. To this aim, SCDMs were allowed to reach complete differentiation in DM for 72 h; then, the myotubes were treated with proNGF for 48 h. ProNGF addition significantly increased fast MyHC expression, whereas slow MyHC levels were strongly reduced, as well as the oxidative marker COX IV. Consistently, AMPK and CREB activating phosphorylations, together with PGC-1α protein expression, were suppressed upon proNGF administration in differentiated myotubes (Figure 6A,B). In order to investigate whether the effects induced by proNGF were mediated by p75NTR activation, myotubes from SCDMs were co-treated with LM11A-31, a non-peptide modulator which blocked proNGF binding to and the activation of p75NTR [40]. In line with our hypothesis, co-treatment with LM11A-31 completely prevented proNGF-induced changes in slow and fast MyHC, cytochrome C, p-CREB, p-AMPK and PGC-1α (Figure 6C,D).

**Figure 7:**
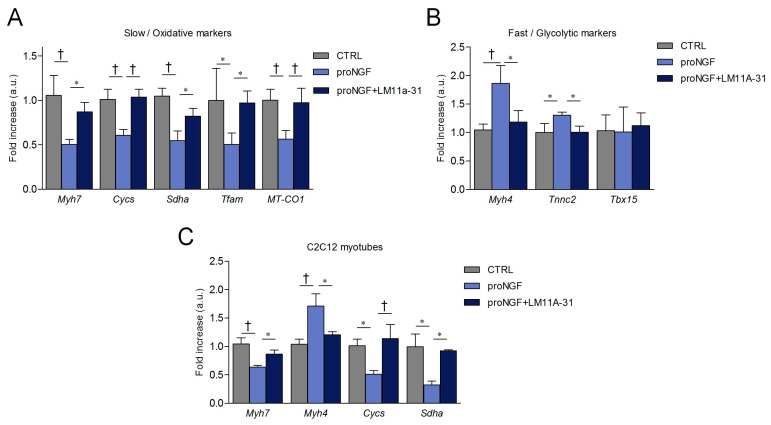
Effects of the proNGF/p75NTR pathway on mRNA levels of slow/oxidative and fast/glycolytic markers in the differentiated myotubes. qRT-PCR analysis of (**A**) the slow/oxidative markers and of (**B**) the fast/glycolytic markers in differentiated myotubes treated with proNGF or proNGF + LM11A-31. SCDMs were differentiated into myotubes for 72 h, and then treated with vehicle (PBS), proNGF (100 ng/mL) and proNGF (100 ng/mL) + LM11A-31 (100 nM) for 48 h. (**C**) C2C12 myotubes were treated as described above, and the transcript levels were evaluated by qRT-PCR. mRNA levels of slow myosin heavy chain 7 (*Myh7*), cytochrome C (*Cycs*), succinate dehydrogenase (*Sdha*), mitochondrial transcription factor A (*Tfam),* cytochrome c oxidase I (*MT-COI*), fast myosin heavy chain 2B (*Myh4*), troponin C2 (*Tnnc2*) and T-box transcription factor 15 (*Tbx15*) are reported as an increase with respect to the control (CTRL) cells treated with vehicle (PBS). Data are normalized against *hprt*, chosen as the reference gene. Statistical analysis was performed by using one-way ANOVA. * *p* < 0.05; ^†^ *p* < 0.01. Arbitrary units (a.u.).

To assess the possibility that other fiber type specific markers were also modulated by proNGF/p75NTR pathway, we estimated the expression of oxidative and glycolytic genes by qRT-PCR analysis in myotubes from SCDMs. We found that proNGF elicited a significant reduction in the transcript levels of genes involved in the slow/oxidative phenotype, such as *Myh7, Cycs, Sdha, Tfam,* and *MT-CO1* (Figure 7A). Differently, pro-neurotrophin addition elevated the mRNA levels of the fast/glycolytic markers *Myh4* and *Tnnc2*, with the exception of *Tbx15*, a transcriptional regulator involved in glycolytic fiber identity (Figure 7B). Importantly, LM11A-31 co-administration efficiently blocked all the alterations induced by proNGF. Comparable results were obtained by analyzing *Myh7, Myh4, Cycs* and *Sdha* expression in differentiated C2C12 myotubes (Figure 7C), suggesting that the activity of the proNGF/p75NTR axis on fiber type specification was conserved in different cells with myogenic potential.

### 3.5. ProNGF and p75NTR Levels Are Increased in Dystrophic Muscles, and Are Accompanied by the Reduction in Markers of Slow/Oxidative Phenotype

**Figure 8:**
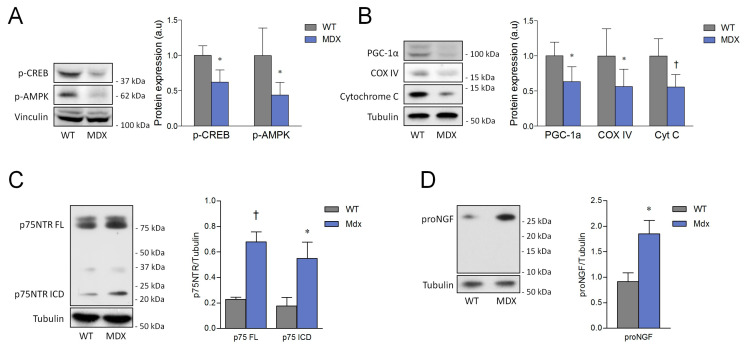
Reduction in the slow/oxidative markers is accompanied by increased expression of proNGF/p75NTR axis in Tibialis anterior (TA) lysates. (**A**) Western blot and densitometric analysis of CREB phosphorylation at Ser133 and of AMPK phosphorylation at Thr172 in three month-old wild-type (WT, N = 3) and mdx mice (N = 6). (**B**) Western blot and densitometric analysis of PGC-1α, COXIV and cytochrome C from three month-old WT (N = 6) and mdx mice (N = 6). (**C**) Immunoblot and densitometric analysis of p75NTR in TA from three month-old WT (N = 3) and mdx mice (N = 4). (**D**) Immunoblot and densitometric analysis of proNGF in TA from three month-old WT (N = 3) and mdx mice (N = 4). Tubulin and vinculin served as the loading control. Data are expressed as the mean ± SD. Statistical analysis was performed by using the Student’s *t* test. * *p* < 0.05; ^†^ *p* < 0.01. Arbitrary units (a.u.).

Dystrophin loss is the leading cause of genetic muscle wasting diseases. It is characterized by membrane fragility and muscle damage, which results in the massive activation of satellite cells in the unsuccessful attempt to counteract fiber degeneration [41,42]. In addition, recent reports highlight that oxidative metabolism is profoundly compromised in dystrophic muscles, and these changes are paralleled by a concurrent reduction in the expression of slow-twitch fibers [41,43,44,45]. Since our results and literature data suggest that the proNGF/p75NTR pathway can be involved in myogenesis and in the regulation of muscle metabolism, we performed Western blot and morphological analyses on the *Tibialis anterior* (TA) of a 3 month-old male mdx mouse, a widely used preclinical experimental model of DMD. The activating phosphorylations of CREB and AMPK resulted to be significantly downregulated in mdx muscles if compared to control (wild-type, WT) animals (Figure 8A). Coherently with the decrease in signaling proteins involved in the oxidative metabolism, PGC-1α and the mitochondrial markers COX IV and cytochrome C were also reduced in dystrophic muscles (Figure 8B). These changes were accompanied by a sustained expression of p75NTR (both the full-length and the ICD fragment) and of proNGF in TA from mdx mice (Figure 8C,D). It is interesting to note that, similarly to what was observed in SCDMs, the mature form of NGF was not detected in the muscle lysates from both wild-type and mdx animals, further sustaining that proNGF is the predominant NGF species in skeletal muscle.

**Figure 9:**
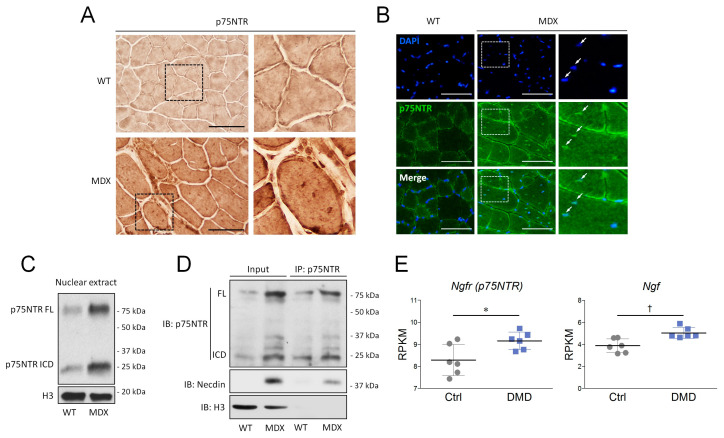
The p75NTR localization and expression in dystrophic muscles from mdx and Duchenne muscular dystrophy (DMD) patients. (**A**) Overview of p75NTR in cross-sections of TA muscles from wild-type (WT) and mdx mice. N = 3 for each experimental group. Scale bar: 100 μm. (**B**) Immunofluorescence analysis of p75NTR (green) expression in TA muscles from WT and mdx animals. DAPI was used to counterstain the nuclei. Nuclear co-localization between p75NTR immunoreactivity and DAPI was indicated by white arrows. N = 3 for each experimental group. Scale bar: 100 μm. (**C**) Western blot of p75NTR in the nuclear fraction from WT and mdx muscles. H3 was used as the protein loading control for the nuclear extract. (**D**) Western blot showing p75NTR/necdin interaction by co-immunoprecipitation experiments. Nuclear extract from WT and mdx muscles were incubated with anti-p75NTR antibody produced in rabbit. Immunoprecipitation was then analyzed for p75NTR and necdin expression by using anti-p75NTR antibody produced in mouse and anti-necdin produced in rabbit. Anti-Rabbit IgG HRP “True blot” were used to visualize co-immunoprecipitated necdin. H3 was used as protein loading control for nuclear extracts. (**E**) RPKM expression levels of *ngfr* (p75NTR) and of *ngf* transcripts in previously reported RNA-Seq dataset from healthy individuals (Ctrl, N = 6) and DMD patients (N = 6). Data are expressed as the mean ± SD. Statistical analysis was performed by using the Student’s *t* test. * *p* < 0.05; ^†^ *p* < 0.01. FL = full-length; ICD = intracellular domain.

Dystrophic muscles are characterized by the infiltration of fibrotic tissue, adipocytes and inflammatory cells [46]. In order to ascertain that changes in p75NTR expression were ascribable to muscle fibers, we performed an immunohistochemical analysis. As observable in Figure 9A, muscle fibers from WT mice showed appreciable p75NTR staining in the cytoplasm, and a stronger immunoreactivity at the membrane level. Consistently with the Western blot results, remarkably higher immunostaining intensity was detected in TA muscle fibers from mdx mice (Figure 9A). Interestingly, mdx muscles also showed densely immunopositive dots inside the cytoplasm, reminiscent of the peculiar centrally located nuclei found in regenerating fibers. Immunofluorescence confirmed this hypothesis, demonstrating a nuclear p75NTR expression as testified by DAPI co-localization (Figure 9B). The p75NTR nuclear localization was further assessed by Western blot analysis on the TA nuclear fraction: both p75NTR FL and p75NTR ICD were expressed in the myonuclei from WT mice, and their expression was significantly increased in mdx muscles (Figure 9C). It has been reported that necdin, an adaptor protein of p75NTR, promotes muscle regeneration by mediating myoblast survival and differentiation [47]. In this context, immunoprecipitation experiments demonstrated that, while absent in WT mice, p75NTR/necdin interaction is strongly enhanced in the myonuclear fraction of mdx muscles, suggesting that this protein complex may be required for muscle regeneration (Figure 9D).

Lastly, in order to evaluate whether changes in the proNGF/p75NTR axis observed in mdx mice were also recapitulated in the muscles from DMD patients, we interrogated RNA-Seq data published by Khairallah and colleagues [48], and found that both *ngf* and *p75NTR* transcript levels significantly increased in DMD muscles when compared to control individuals (Figure 9E). The rise of *ngf* and *p75NTR* transcripts was also paralleled by the reduction of the slow/oxidative markers, such as *Cycs*, *COXIV*, *Ppargc1a*, *Myh2* and *Myh7*; differently, no changes were observed for the expression of the fast *Myh4* (Figure S2A,B). Taken together, these results underline that dystrophic muscles are characterized by an increased expression of both proNGF and p75NTR, which are accompanied by disbalances in the expression of oxidative metabolism markers.

## 4. Discussion

Neurotrophins are best known for their incontrovertible role in the regulation of several biological processes in brain cells. However, in recent years, increasing evidence highlights that this class of neurotrophic factors may exert important physiological functions in other cell contexts [1]. For instance, skeletal muscle attracted interest as a considerable source of neurotrophins, especially during development. Neurotrophins produced by skeletal muscle may act as autocrine/paracrine factors within the muscle compartment, as skeletal muscle cells express appreciable amounts of neurotrophin receptors [1,49]. Among neurotrophins, BDNF is the most studied in the neuromuscular system. Indeed, this growth factor exerts pivotal physiological roles, facilitating the survival and the innervation pattern of motor neurons during development, regulating muscle regeneration, and affecting muscle strength [10,12,50]. Furthermore, recent findings also demonstrated the crucial involvement of BDNF signaling in the regulation of skeletal muscle metabolism and fiber type specification [12,51]. Concerning NGF, different studies highlighted a role in skeletal muscle physiology, with particular reference to the ability of this neurotrophin to modulate the regenerative capacity of myogenic precursors through the low-affinity receptor p75NTR [13,14,16,19]. Despite this evidence, information about the influence of NGF signaling on skeletal muscle homeostasis is still limited, and no studies systematically addressed the prospective role of NGF in the regulation of skeletal muscle phenotype.

In this work, we aimed at better characterizing the putative role exerted by NGF/p75NTR signaling in the regulation of skeletal muscle metabolism. To start, we corroborated previous findings [16], observing that p75NTR was expressed in proliferating SCDMs, and its levels tended to increase when the cells were cultured in the differentiation medium. Notably, we noted for the first time that p75NTR also localized at the nuclear level, both in SCDMs and in centrally located nuclei of mdx muscles. Concurrently, we performed immunoblot analysis by using an antibody able to recognize both the precursor (proNGF) and the mature NGF. The results show that proNGF species is the predominant form of NGF expressed in SCDMs and TA muscles, leading us to hypothesize that the proNGF/p75NTR axis may exert a role in skeletal muscle physiology. When we added exogenous proNGF, no effects were observed in the ability of SCDMs to proliferate or differentiate. On the contrary, when an endogenous proNGF was inactivated by anti-NGF administration, a slight decrease in SCDMs proliferation and a significant decrease in myotube differentiation was noted. Importantly, this is the first report aimed at assessing the effects of proNGF on the proliferation and differentiation of myogenic precursors since, to our knowledge, literature data only illustrated the putative biological activity of mature NGF administration [16,17]. Nevertheless, comparable results were obtained by Deponti and colleagues, demonstrating that the disruption of p75NTR signaling by blocking the peptide effectively decreases myotube fusion, whereas the addition of exogenous mature NGF does not enhance the myogenic process [16]. Taken together, these results indicate that p75NTR activation by the endogenous neurotrophin is already able to induce a maximal effect on SCDM differentiation.

However, proNGF treatment is not exempt from biological activity on differentiating SCDMs. Notably, the addition of exogenous proNGF hindered the time-dependent increase in slow/oxidative markers, and significantly promoted the expression of fast MyHC during myogenic differentiation. Importantly, proNGF stimulation was not only capable of driving SCDM metabolism during differentiation, as it also mediated a powerful fiber shift in mature myotubes. In this context, exogenous proNGF administration strongly reduced the expression of slow/oxidative markers and facilitated the induction of a fast-twitch glycolytic muscle fiber program. The effects produced by proNGF treatment are mediated by p75NTR activation. Indeed, p75NTR blockade by LM11A-31 completely prevented the rise in the fast MyHC and in the glycolytic marker Troponin-C fast (*tnnc2*). Concurrently, p75NTR inhibition also counteracted the proNGF-mediated reduction of targets involved in the slow/oxidative program. Biochemical analysis revealed that the proNGF/p75NTR axis may suppress slow fiber type specification by mitigating AMPK and CREB activation. The metabolic sensor AMPK is a key regulator of mitochondrial biogenesis, and its activity is strictly connected with the establishment of a slow phenotype with high oxidative capacity [38,52]. On the other hand, the activation of the transcription factor CREB is crucial for the induction of genes involved in the oxidative metabolism, including cytochrome C and COX IV [39]. It is well established that both AMPK and CREB signaling converge in the promotion of the slow/oxidative program, at least in part by positively regulating the expression of the transcriptional coactivator PGC-1α [38,39]. Interestingly, the proNGF-dependent decrease in the activating phosphorylation of CREB and AMPK was also paralleled by a significant reduction in PGC-1α expression, which was completely prevented by p75NTR modulation though LM11A-31.

Recently, an interesting research linked p75NTR to energy balance in adipose tissue. In particular, p75NTR was shown to directly interact with the catalytic subunit of protein kinase A (PKA), affecting CREB activity and PGC-1α expression in adipocytes [53]. In the light of these observations, we speculated that proNGF may potentiate the interaction between p75NTR and PKA, leading to the suppression of the PKA/CREB/PGC-1α pathway and the subsequent occurrence of the metabolic shift observed in this work.

The homeostatic maintenance of energy metabolism is an essential prerequisite for the proper functionality of muscle fibers. For this reason, it is not surprising that recent studies pointed out that pathological conditions, such as DMD, are characterized by severe alterations in the oxidative metabolism: notably, reduced ATP content and low a mitochondrial number contributes to the onset of a “metabolic crisis”, which affects the ability of the dystrophic muscle to respond to metabolic requirements [41,43]. The expression of the slower MyHC isoforms is also reduced in both mdx mice and DMD individuals. For instance, Ljubicic and colleagues highlighted that TA muscles from mdx mice are characterized by a marked decrease in MyHC IIa; whereas other studies provided evidence for lower MyHC I in muscles from DMD individuals [44,45]. Coherently, in the present work, we observed a decrease in the mitochondrial markers, which was paralleled by a significant suppression in CREB and AMPK activation in TA muscles from mdx mice. The interrogation of RNA-seq data published by Khairallah and colleagues [48] corroborates these findings, as the transcript levels of slow/oxidative markers such as COX IV, cytochrome C, PGC-1α, Myh2 and Myh7 were found to be downregulated also in DMD muscle biopsies. Importantly, the concurrent buildup of proNGF/p75NTR protein levels in mdx muscles, together with the increase in *ngf/ngfr* transcripts in DMD patients, suggest that the hyperactivation of the proNGF/p75NTR pathway could counteract the establishment of the slow/oxidative phenotype in the dystrophic pathology, in a similar fashion to that observed in SCDMs. We also unveiled that p75NTR shows nuclear localization in both SCDMs and TA muscles. It has been reported that, upon proNGF activation, p75NTR may translocate into the nucleus and interact with adaptor proteins to regulate signaling cascades, nucleocytoplasmic shuttling and transcriptional activation [31,54,55]. To our knowledge, this is the first report demonstrating that p75NTR nuclear localization also occurs in muscle cells. The p75NTR expression is particularly prominent in centrally located nuclei from regenerating fibers of mdx mice, hypothesizing that the nuclear translocation of the receptor could participate in the modulation of muscle regeneration in the dystrophic phenotype. This assumption is further strengthened by the fact that p75NTR interaction with necdin is strongly enhanced in nuclear extracts from mdx muscles. Literature data already provided evidence for a crucial role of necdin in myogenic differentiation and muscle regeneration in numerous physiopathological conditions [47,56,57,58]. Remarkably, necdin levels are undetectable in adult muscle and high in during myogenic differentiation, in agreement with the notion that the expression of this protein is exclusively restricted during active muscle differentiation [47]. Since necdin recruitment is crucial for assuring several p75NTR signaling properties, we speculated that p75NTR/necdin interaction into the nuclear compartment may participate in the control of the regenerative process occurring in the dystrophic phenotype. The buildup of proNGF and p75NTR protein expression in mdx muscles is further supported by RNA-Seq results performed on skeletal muscles from DMD individuals, showing that both *ngfr* (the gene codifying for p75NTR) and *ngf* transcripts are significantly increased.

Despite other research being mandatory to reach an in-depth comprehension of the molecular mechanisms linking the proNGF/p75NTR axis and muscle biology, our results highlighted that the proNGF/p75NTR pathway may play a relevant role in muscle physiology, as the basal activation of p75NTR signaling is required for proper myogenic differentiation. Moreover, enhanced p75NTR activation by exogenous proNGF addition affects fiber type determination, thus facilitating the occurrence of a fast-twitch phenotype. These events suggest that physiopathological conditions characterized by reinforced proNGF/p75NTR signaling may develop substantial metabolic alterations in skeletal muscle. Other efforts should be done to evaluate the effective contribution of this pathway in muscle physiology and pathology. Indeed, it is now well established that slower and oxidative muscle fibers are more resistant to the dystrophic damage when compared to the faster/glycolytic fibers [59], and phenotypic modifiers implicated in the fast-to-slow shift of skeletal muscle fibers represent promising targets for prospective pharmacological approaches against DMD [44]. Whether successfully translated in in vivo studies, our findings could set the basis for innovative therapies based on p75NTR targeting for counteracting or alleviating symptoms associated with dystrophin deficiency. Furthermore, the use of the p75NTR inhibitor LM11A-31 as prospective pharmacological agent may have strong relevance in terms of clinical implications. Notably, in a Phase I trial, LM11a-31 administration at single or multiple doses was well tolerated by young or old volunteers with no important adverse events and is currently employed in an ongoing phase II clinical trial for Alzheimer’s disease (NCT03069014).

## Supplementary Materials

The following are available online at https://www.mdpi.com/2073-4409/9/10/2232/s1, Figure S1. Validation of anti-NGF (E-12, sc-365944, Santa Cruz Biotechnology) in the hippocampi of wild-type and mdx mice. Western blot showing a clear band at approximately 26 kDa, corresponding to proNGF, and a second band at approximately 13.5 kDa, corresponding to mature NGF. Actin was chosen as the housekeeping protein. Mouse brain hippocampus was chosen as the positive control for the detection of both the precursor and mature form of NGF. Figure S2. (A) RPKM expression levels of *Myh7*, *Myh2*, and *Myh4and;* (B) RPKM expression levels of the oxidative markers PGC-1α (*Ppargc1a*), *COX IV* and cytochrome C (*Cycs*) from the RNA-Seq dataset previously published by Khairallah et al. Healthy individuals (Ctrl, N = 6) and DMD patients (N = 6). Data are expressed as the mean ± SD. Statistical analysis was performed by using the Student’s *t* test. * *p* < 0.05; ^†^ *p* < 0.01; ^‡^ *p* < 0.001. Table S1. List of primers used in quantitative RT-PCR analysis.
