# Supplementary material for: ProNGF/p75NTR Axis Drives Fiber Type Specification by Inducing the Fast-Glycolytic Phenotype in Mouse Skeletal Muscle Cells
Source: Cells. 2020 Oct 2;9(10):2232. doi: 10.3390/cells9102232 (PMC7599914; doi:10.3390/cells9102232)
Supplement: Supplementary file 1 [file cells-09-02232-s001.pdf]

Supplementary Materials

Supplementary Table S1

Table S1. List of primers used in quantitative RT-PCR analysis		
Mouse gene	***Forward***	***Reverse***
Hprt	5’–CCTCATGGACTGATTATGGACAG	5’–TCAGCAAAGAACTTATAGCCCC
Myh7	5’-GCCAACTATGCTGGAGCTGATGCCC	5’-GGTGCGTGGAGCGCAAGTTTGTCATAAG
Myh4	5’-GAGCTACTGGATGCCAGTGAGCGC	5’-CTGGACGATGTCTTCCATCTCTCC
Tnnc2	5’-CCATCATCGAGGAGGTGGAC	5’-CTTCCCCTTCGCATCCTCTT
Tbx15	5’-TGTTCGCACACTGACCTTTG	5’-CCAGTGCTGGAGGTGGTT
MtCO1	5’-TGCTAGCCGCAGGCATTAC	5’-GGGTGCCCAAAGAATCAGAAC
Sdha	5’-GCTGGTGTGGATGTCACTAAGG	5’-CCCACCCATGTTGTAATGCA
Cycs	5′-GCAAGCATAAGACTGGACCAAA	5′-TTGTTGGCATCTGTGTAAGAGAATC
Tfam	5’-AAGGGAATGGGAAAGGTAGA	5’-AACAGGACATGGAAAGCAGAT

Supplementary Figure S1

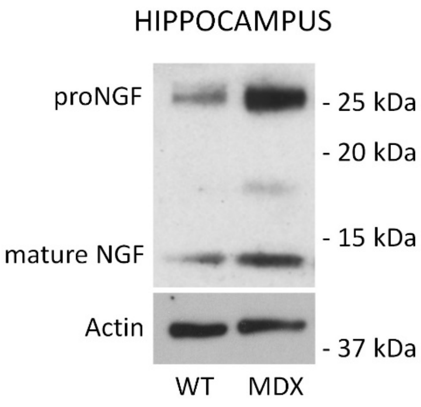

Supplementary Figure 1. Validation of anti-NGF (E-12, sc-365944, Santa Cruz Biotechnology) in the hippocampi of wild type and mdx mice. Western blot showing a clear band at approximately 26 kDa, corresponding to proNGF, and a second band at approximately 13.5 kDa, corresponding to mature NGF. Actin was chosen as housekeeping protein. Mouse brain hippocampus was chosen as positive control for the detection of both precursor and mature form of NGF.

Supplementary Figure S2

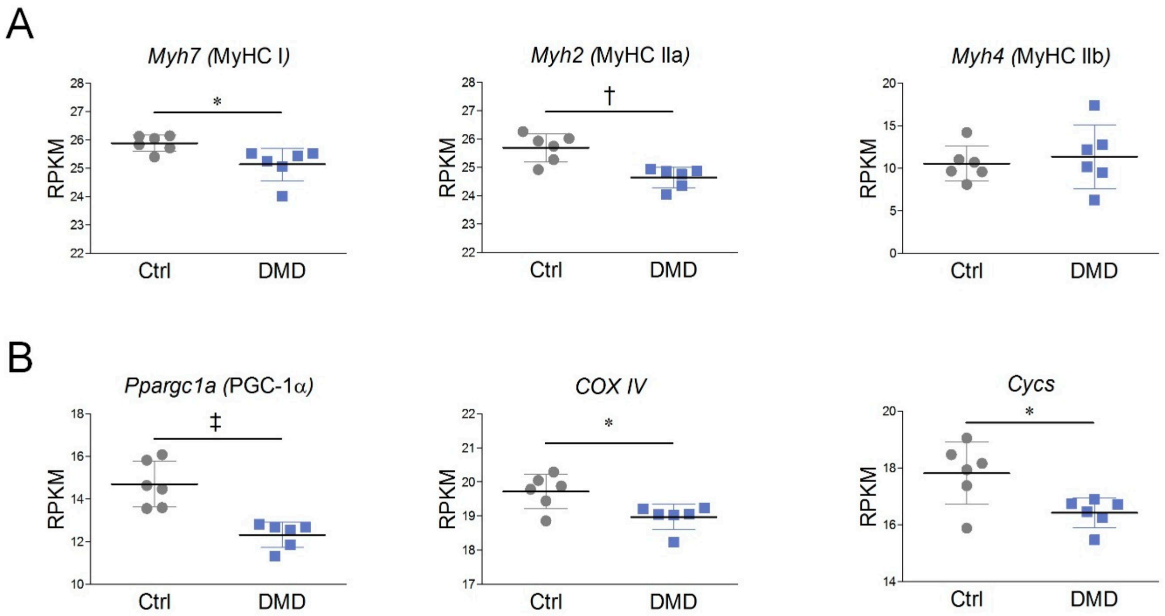

Supplementary Figure 2. (A) RPKM expression levels of *Myh7*, *Myh2*, and *Myh4* and (B) RPKM expression levels of the oxidative markers PGC-1α (*Ppargc1a*), *COX IV* and cytochrome C (*Cycs*) from RNA-Seq dataset previously published by Khairallah et al. Healthy individuals (Ctrl, N=6) and DMD patients (N=6). Data are expressed as mean±SD. Statistical analysis was perfomed by using Student's t test. *P<0.05; †P<0.01; ‡P<0.001.
